# Temperature and elevated CO_2_ alter soybean seed yield and quality, exhibiting transgenerational effects on seedling emergence and vigor

**DOI:** 10.3389/fpls.2024.1427086

**Published:** 2024-07-31

**Authors:** Naflath Thenveettil, Raju Bheemanahalli, Krishna N. Reddy, Wei Gao, K. Raja Reddy

**Affiliations:** ^1^ Department of Plant and Soil Sciences, Mississippi State University, Mississippi State, MS, United States; ^2^ United States Department of Agriculture - Agricultural Research Service (USDA-ARS), Crop Production Systems Research Unit, Stoneville, MS, United States; ^3^ United States Department of Agriculture - Ultraviolet B (USDA UVB) Monitoring and Research Program, Department of Ecosystem Science and Sustainability, Colorado State University, Fort Collins, CO, United States

**Keywords:** CO_2_, seed yield, seed quality, seedling vigor response index, temperature, transgenerational effect

## Abstract

**Introduction:**

Environmental conditions play a prime role in the growth and development of plant species, exerting a significant influence on their reproductive capacity. Soybean is sensitive to high temperatures during flowering and seed developmental stages. Little is known about the combined environmental effect of temperature and CO_2_ on seed yield and quality and its future generation.

**Methods:**

A study was conducted to examine the effect of temperature (22/14°C (low), 30/22°C (optimum), and 38/30°C (high)), and CO_2_ (420 ppm (ambient; aCO_2_) and 720 ppm (elevated; eCO_2_)) on seed yield, quality, and transgenerational seedling vigor traits of soybean cultivars (DS25-1 and DS31-243) using Soil-Plant-Atmospheric-Research facility.

**Results:**

A significant temperature effect was recorded among yield and quality attributes. At high-temperature, the 100-seed weights of DS25-1 and DS31-243 declined by 40% and 24%, respectively, over the optimum temperature at aCO_2_. The harvest index of varieties reduced by 70% when exposed to high temperature under both aCO2 and eCO_2_, compared to the optimum temperature at aCO_2_. The seed oil (- 2%) and protein (8%) content altered when developed under high temperature under aCO_2_. Maximum sucrose (7.5%) and stachyose (3.8%) accumulation in seeds were observed when developed under low temperatures and eCO_2_. When the growing temperature increased from optimum to high, the seed oleic acids increased (63%), while linoleic and linolenic acids decreased (- 28% and - 43%, respectively). Significant temperature and CO_2_ effects were observed in progenies with the highest maximum seedling emergence (80%), lesser time to 50% emergence (5.5 days), and higher seedling vigor from parents grown at low-temperature treatment under eCO_2_.

**Discussion:**

Exposure of plants to 38/30°C was detrimental to soybean seed yield, and eCO_2_ levels did not compensate for this yield loss. The high temperature during seed developmental stages altered the chemical composition of the seed, leading to an increased content of monounsaturated fatty acids. The findings suggest that parental stress can significantly impact the development of offspring, indicating that epigenetic regulation or memory repose may be at play.

## 1 Introduction

Climate change is an ongoing process, and the Industrial Revolution has substantially intensified the challenges confronting Earth’s climate (NOAA, 2021). The trajectory of CO_2_ levels in the atmosphere shows that the beginning of the industrial age increased the abundance of CO_2_ by around 45% (Buis, 2019), bringing it up to 420 ppm. The global surface temperature has risen by 1.09°C in the last decade compared to 1850-1900, with a faster increase since 1970 than any other 50-year window during the previous 2000 years (Pachauri et al., 2014; Calvin et al., 2023). Due to these climate changes, the ten hottest years occurred in the last 15 years, and the summer of 2023 is confirmed to be the warmest on record (Copernicus Climate Change Service, 2023). In addition, the climate models project global differences in temperature means and extremes if global warming reaches 1.5°C (IPCC, 2018). Recent studies have highlighted that developed countries exhibit higher vulnerability (8-11%) to climate change than developing countries (Lesk et al., 2016; Altieri and Nicholls, 2017). Over the years, global extreme climate events have increased, causing an economic loss of over 220 billion USD in 2022 (CRED, 2023).

Environmental conditions play a prime role in the growth and development of plant species, exerting a significant influence on their reproductive capacity (Raza et al., 2019). Among the critical abiotic factors, temperature, water, and light play a crucial role in shaping plant physiology and reproductive processes. Temperature beyond 30°C can adversely affect plant growth and physiology, leading to a reduction in photosynthesis, flowering, and seed development, ultimately resulting in yield loss (Hatfield and Prueger, 2015). Conversely, sub-optimal temperatures can impede germination and growth rates, hampering overall plant development (Szczerba et al., 2021). Given the concurrent likelihood of changes in CO_2_ and temperature, it is critical to quantify the interactions between these two climate variables. While enhanced photosynthetic and agronomic growth has been reported under eCO_2_, accompanied by reduced water use efficiency and nitrogen and protein accumulation (Taub, 2010), the specific effects under varying temperature conditions remain understudied.

Soybean is an important crop with a global production of 399.5 million metric tons (USDA-FAS, 2023). The U.S. stands second in world soybean production after Brazil, encompassing 86.3 million acres (MSPB, 2023) and boasting a U.S. export value of 34.37 billion USD (USDA-FAS, 2023). The crop is widely utilized in food, livestock, and industrial sectors due to its high nutritional value and contributes 90% of the U.S. oil seed production. The optimal temperature of soybeans is estimated to be around 25-30°C (Alsajri et al., 2020), and any deviation has the potential to impact plant growth.

Given the relatively lower optimal temperature (26°C) for anthesis and seed development compared to the vegetative stage (30°C), the reproductive stage is the most critical and thermally sensitive phase for soybean development (Hatfield et al., 2011). Consequently, comprehending the temperature-induced responses in soybean becomes imperative for the development of effective mitigation strategies. A study by Tacarindua et al. (2013) reported that a 3°C rise from the ambient temperature during flowering to the early seed-filling stage in soybean led to a 27% reduction in dry matter accumulation, accompanied by decreased photosynthesis and stomatal conductance. Similarly, cold stress during the flowering stage in soybean leads to the non-opening of flowers, leading to inadequate pollination and subsequent lower pod set or production of barren pods (Ohnishi et al., 2010). Temperature fluctuations also impact seed quality, with documented instances of decreased protein and increased oil accumulation (Bellaloui et al., 2015; Mourtzinis et al., 2017; Poudel et al., 2023).

Over the past century, the average annual temperature in soybean growing regions rose1°C (Zhao et al., 2017), resulting in a 26% yield reduction (Sharma et al., 2022), emphasizing heightened concerns for the reproductive and seed-filling stages in soybean. While plants exhibit direct responses to the growing environment, their phenotypes can also be influenced by ancestral environmental conditions (Uller, 2008). Existing research underscores that certain environmental stress effects can be transmitted to progenies, adversely impacting their growth and development (Tielborger and Petru, 2010; Cendán et al., 2013). Numerous studies on *Arabidopsis thaliana* suggest that plant’s transgenerational memory plays a crucial role in delivering stress-induced responses, with offspring adaptation to specific stress conditions influenced by the parent’s stress response (Suter and Widmer, 2013). Wijewardana et al. (2019) demonstrated the transgenerational impact of drought stress in soybeans, which led to reduced maximum seed germination, seed germination rate, and overall seedling performance in the F_1_ generation. Similarly, Alsajri et al. (2022) reported a reduced seed germination rate in progenies under extreme growth temperatures. To date, no information is available on the transgenerational effects of temperature and CO_2_ on soybean.

Furthermore, limited studies have been dedicated to understanding the relationship between seed composition and seedling performance. It has been proposed that increased seed mass will better support the early seedling establishment (Zas et al., 2013). In this study, we looked at the individual seed-to-seedling dynamics within the framework of seed chemical composition, with a specific focus on elucidating the correlation between per-seed composition and the growth and development of seedlings. Therefore, the present study hypothesized that the two soybean cultivars possessing heat and drought-tolerant traits differentially respond to temperature and CO_2_ stress and that the effect of these stresses is transgenerational. Additionally, we also postulate that the per-seed chemical composition determines the seedling vigor. The objectives of the study were (1) to quantify the effect of temperature and CO_2_ on soybean seed yield and quality, (2) to determine the transgenerational effect of temperature and CO_2_ on seed emergence and seedling vigor, and (3) to draw the relationship between per-seed chemical composition and seedling vigor.

## 2 Materials and methods

The study was conducted in the Soil-Plant-Atmosphere-Research (SPAR) facility at the Environmental Plant Physiology Laboratory, Mississippi State University, MS, USA, from 2022 to 2023. The SPAR chambers precisely manipulate and monitor the CO_2_ and temperature through an automated control system (**Supplementary Figure 1**). At the same time, the plants are being grown under natural sunlight conditions, unlike other controlled environmental growth chambers. The units contain a metal bin and a chamber with Plexiglass (1.27 cm thickness) housing the plant canopy, permitting 97% visible solar radiation. A heating and cooling system controls the temperature inside the chamber. The CO_2_ levels are monitored by a CO_2_ analyzer and adjusted through the control system. The details and specifications of the SPAR units have been presented by Reddy et al. (2001).

### 2.1 Experimental details

The study selected heat-tolerant (DS25-1) and drought-tolerant (DS31-243) soybean cultivars with indeterminate growth habits belonging to maturity Group IV. The research comprised two distinct experiments designed to comprehensively examine the effects of temperature and CO2 on soybean seed yield and quality and the influence of these treatments on the subsequent performance of the progeny.

#### 2.1.1 Experiment I: temperature and CO_2_ effect on seed yield and quality of soybean

##### 2.1.1.1 Experimental setup

Seeds of DS25-1 and DS31-243 were sown in polyvinyl chloride (PVC) pots (15 × 46 cm; 8-liter capacity) filled with a mixture of a 3:1 ratio of fine sand and topsoil by volume (87% sand, 2% clay, and 11% silt). The pots consisted of a hole of 1 cm diameter at the bottom and were filled with 250g of gravel to facilitate easy drainage. Twenty pots were arranged in 10 columns × 2 rows within each SPAR unit, accommodating 10 pots for each cultivar. Four seeds of two cultivars were sown in alternative columns for randomization. The plants were thinned to one plant per pot at the two-leaf stage. The units were maintained at day/night temperatures of 30/22°C. In contrast, the CO_2_ of three SPAR units was maintained at 420 ppm, and another three units were maintained at 720 ppm to imitate the future environment. The pots were irrigated thrice a day with full-strength modified Hoagland’s nutrient solution (Hewitt, 1953) for an adequate supply of water and nutrients. Throughout the experiment, the irrigation volume was adjusted according to the daily evapotranspiration (Reddy et al., 2001).

##### 2.1.1.2 Treatments

At the time of flowering (40 days after sowing), the temperature treatments were imposed in each SPAR unit. Three day/night temperatures of 22/14°C (low), 30/22°C (optimum), and 38/30°C (high) were maintained in each SPAR unit at two CO_2_ concentrations of 420 ppm (aCO_2_) and 720 ppm (eCO_2_). The experiment was carried out in a three-factorial, completely randomized design, considering CO_2_, temperature, and cultivar as the main source of variation. The selection of high and low temperatures was based on the previous reports of the optimum temperature of soybean (Alsajri et al., 2020). The daytime temperatures were maintained from sunrise to sunset, with night-time temperatures transitioning over 30 minutes after sunset. Black netted shade cloths were positioned around the edges of the plexiglass chambers to simulate the impact of border plants. The shade net was regularly raised depending on the plant growth.

##### 2.1.1.3 Observations

All the plants were harvested 120 days after planting, corresponding to 80 days after the initiation of treatments. The seed yield attributes were recorded at the time of harvest. The number of pods (no. plant^-1^) was recorded by counting fully matured pods containing one or more seeds. The pods were hand-threshed after oven-drying at 35°C for 24 hours to maintain a seed moisture content of 14% uniformly. The seeds (no. plant^-1^) were counted using an Old Mill seed counter (NP5056-Model 850-2, LICOR Inc., Lincoln, NE, USA). The 100 seed weight (g) and seed yield (g plant^-1^) were recorded for all the treatments. Following the measurements, the total biomass was recorded by subjecting the plant parts (leaf, stem, pod, and root) to oven drying at 80°C for 72 hours. The resulting weights were expressed in grams. The harvest index was calculated by dividing the seed yield by total biomass.

The harvested seeds from each treatment were analyzed for quality. Five grams of seeds from each of the six treatment combinations were analyzed for protein, oil, fatty acids, and sugars using NIRS spectroscopy (Perten DA7520 spectrometer, Perten Instruments, IL, USA), and the content was expressed in percentage.

#### 2.1.2 Experiment II: transgenerational effect of temperature and CO_2_ in soybean

##### 2.1.2.1 Experimental setup

This experiment was conducted during May 2023, using the seeds harvested from experiment I, hereafter termed progeny, which underwent temperature and CO_2_ alterations during its developmental and maturation stage. The seeds were randomly selected from each treatment and were sown in PVC pots (15 × 46 cm) filled with a 3:1 ratio of fine sand and topsoil mixture. Four seeds were sown in each treatment and were thinned to one plant per pot after the first leaf stage. The pots were arranged in a completely randomized design with four replications per treatment on a concrete platform. The modified Hoagland’s nutrient solution was supplied via an automated drip irrigation system. The plants were grown under ambient conditions of temperature and CO_2_.

##### 2.1.2.2 Observations

The transgenerational effects of temperature and CO_2_ were estimated through progeny performance. The seedling emergence was recorded twice a day at 8.00 and 16.00 hours, considering the seedlings with widely opened cotyledons emerged. After 20 days of sowing, the seedlings were cut at the soil level, and the plant height and leaf area were taken. The plant height was recorded from the base of the stem to the tip of the apical bud. The total leaf area was measured using a LI-3100 leaf area meter (LI-COR, Inc., Lincoln, NE). Plant parts were separated into leaves, stems, and roots, and the biomass was dried at 80°C for 72 hours. The total biomass was calculated by summing the individual biomass values of leaves, stems, and roots and expressed in grams.

### 2.2 Data analysis

Analysis of variance was performed to ascertain the effect of temperature, CO_2_, and cultivars on various growth and yield parameters using three factorial completely randomized design for experiment I and experiment II. The analysis was performed in R studio using ‘doebioresearch’ package (Popat and Banakara, 2022). Multiple comparison tests were carried out to identify cultivar-specific responses to treatment effects at a 0.05 level of significance using a t-test. To determine the impact of temperature and cultivar or CO_2_ and cultivar, a two-way factorial, completely randomized analysis of variance was performed. Graphical representations of the outcomes were generated using Sigmaplot 13.0 (Systat Software Inc., San Jose, CA, USA).

#### 2.2.1 Seedling emergence curve fitting

The germination time course of seeds was fitted in a three-parameter sigmoidal function (Equation 1) using Sigmaplot 13.0 (Singh et al., 2019).


(1) $$Y=\;\frac{MSG}{\left\{1+\;e^{\left[-\frac{t-t_{50}}{G_{rate}}\right]}\right\}}$$

Where ‘Y’ denotes the cumulative seed germination percentage, ‘MSG’ is the maximum seed germination percentage, t_50_ is time to 50% emergence, and G_rate_ is the slope of the curve. Using these parameters, the difference in seedling establishment was estimated. The analysis of variance of maximum seed germination (MSG), seed germination rate (SGR), and t_50_ was performed in R studio using three factorial completely randomized design considering parental treatments such as temperature, CO_2_, and cultivar as the main source of variation.

#### 2.2.2 Seedling vigor response index

The measured and computed parameters of seedling establishment and growth were utilized to calculate the seedling vigor response index. The individual seedling vigor response index (IVRI) of each parameter was computed by dividing the observed value in each treatment (P_i_) by the maximum value from all the treatments (P_m_; Equation 2). However, IVRI for t_50_ was calculated by dividing least value (P_l_) with the individual observed value (Equation 3). The average values were used for the computation. The cumulative vigor response index (CVRI) was computed by summing the IVRI of all the parameters for each treatment combination.


(2) $$IVRI=\frac{P_{i}}{P_{m}}$$

(3) $$IVRI\;\left(t_{50}\right)=\frac{P_{l}}{P_{i}}$$

#### 2.2.3 Seed constitution to seedling performance

To understand the relationship between per-seed chemical composition and the seedling performance, percentage seed quality parameters estimated during experiment I were converted into per-seed basis using the seed weight and were expressed in milligrams per seed (mg seed^-1^). A linear regression analysis (Equation 4) was conducted using these individually calculated per-seed quality components against the CVRI, and the regression correlation coefficient (R^2^) was monitored to determine the model fitness. We also noticed the relationship between seed emergence and seedling vigor to understand the relationship between metrics of seed germination and seedling vigor.


(4) Y=mx+b

Where Y is the dependent variable (CVRI), x is the independent variable (individual seed quality parameters), m is the slope of the line, and b is the y-intercept.

## 3 Results

### 3.1 Temperature and CO_2_ effect on seed yield

The growing temperature during flowering and seed developmental stages significantly impacted (p<0.5 to p<0.001) the seed yield traits (**Table 1**). The effect of eCO_2_ was non-significant for 100-seed weight, seed yield, and harvest index, while there was a detectable difference in pod number and seed number at p<0.05. The two cultivars differed in pod number, 100-seed weight, and seed yield. Among the seed yield traits, only 100-seed weights exhibited notable differences due to the interaction of temperature and CO_2_, as well as temperature and cultivar. All other interactions were deemed non-significant across all recorded yield traits.

**Table 1:** Analysis of variance across temperature (T), CO_2_ and cultivars (V) for seed yield parameters (*, **, *** represent significance level p ≤ 0.05, 0.01, and 0.001, respectively, and NS-non-significant).

Parameters	T	CO_2_	V	T*CO_2_	T*V	CO_2_*V	T*CO_2_*V
Pod (no. plant^-1^)	*	*	*	NS	NS	NS	NS
Seed (no. plant^-1^)	**	*	NS	NS	NS	NS	NS
100 Seed weight (g)	***	NS	***	*	*	NS	NS
Seed yield (g plant^-1^)	***	NS	*	NS	NS	NS	NS
Harvest index	***	NS	NS	NS	NS	NS	NS

**Figure 1:**
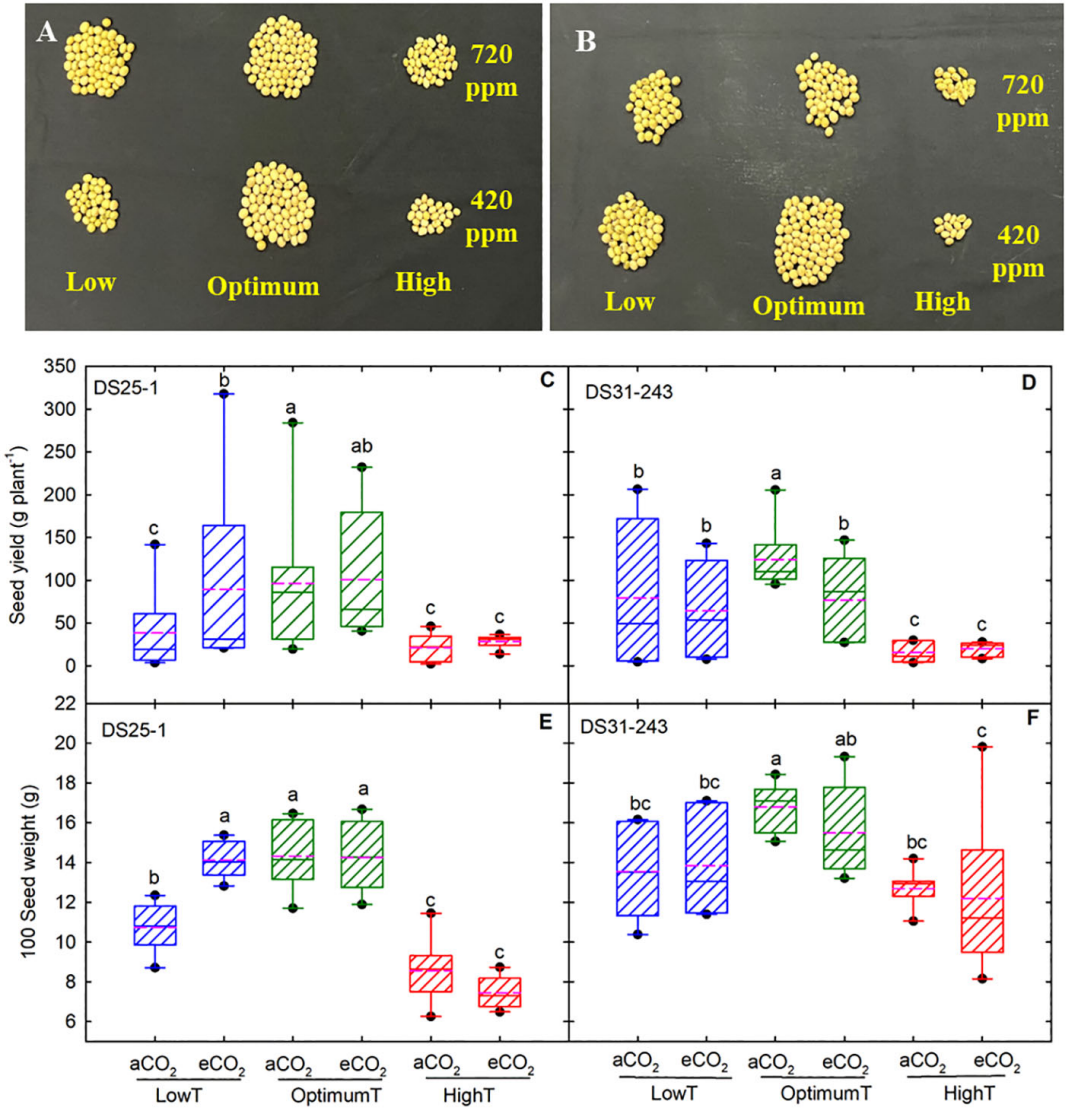
Temperature (LowT: 22/14°C; optimumT: 30/22°C; highT: 38/30°C) and CO_2_ (aCO_2_: 420 ppm and eCO_2_: 720 ppm) effects on seed yield, **(A)** DS25-1, **(B)** DS31-243 (Picture shows 10% of total seed yield), **(C, D)** box plot of seed yield (g plant^-1^), and **(E, F)** 100 seed weight **(G)** of DS25-1 and DS31-243. The pink dotted line indicates the average values. The letters above the boxes are Duncan’s multiple range test (DMRT) between six treatments. The treatment boxes with similar letters are statistically non-significant.

**Figure 2:**
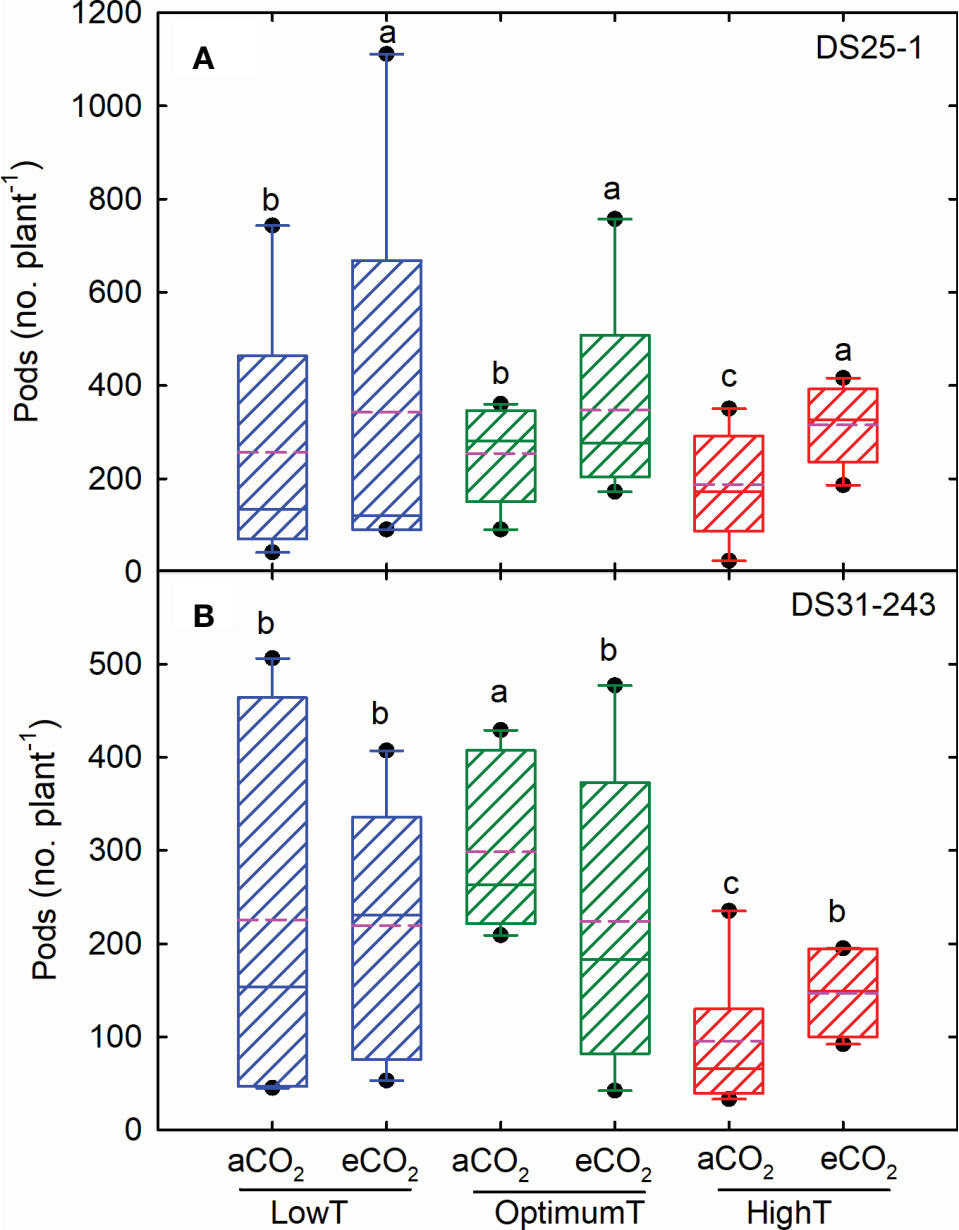
Temperature (LowT: 22/14°C; optimumT: 30/22°C; highT: 38/30°C) and CO_2_ (aCO_2_: 420 ppm and eCO_2_: 720 ppm) effects on pod number of **(A)** DS25-1 and **(B)** DS31-243. The pink dotted line indicates the average values. The letters above the boxes are Duncan’s multiple range test (DMRT) between six treatments. The treatment boxes with similar letters are statistically non-significant.

The high temperature reduced the seed yield and 100-seed weight in both cultivars (**Figures 1**, **2**). The maximum seed yield was observed at optimum temperature. The eCO_2_ did not increase the yield in both cultivars at optimum temperature. The decline in seed yield at high temperatures was highest in DS31-243 (88% at aCO_2_ and 84% at eCO_2_) than DS25-1 (78% aCO_2_ and 70% at eCO_2_) compared to optimum temperature and aCO_2_. The low temperature did not have any significant effect on seed yield in DS25-1 and DS31-243 at eCO_2_. High growing temperature reduced the 100-seed weight of both cultivars without being potentially affected by eCO_2_ (**Figures 1E, F**). The decline in 100-seed weight at high temperatures was highest in DS25-1 (40% at aCO_2_ and 47% at eCO_2_) than in DS31-243 (24% at aCO_2_ and 23% at eCO_2_).

**Figure 3:**
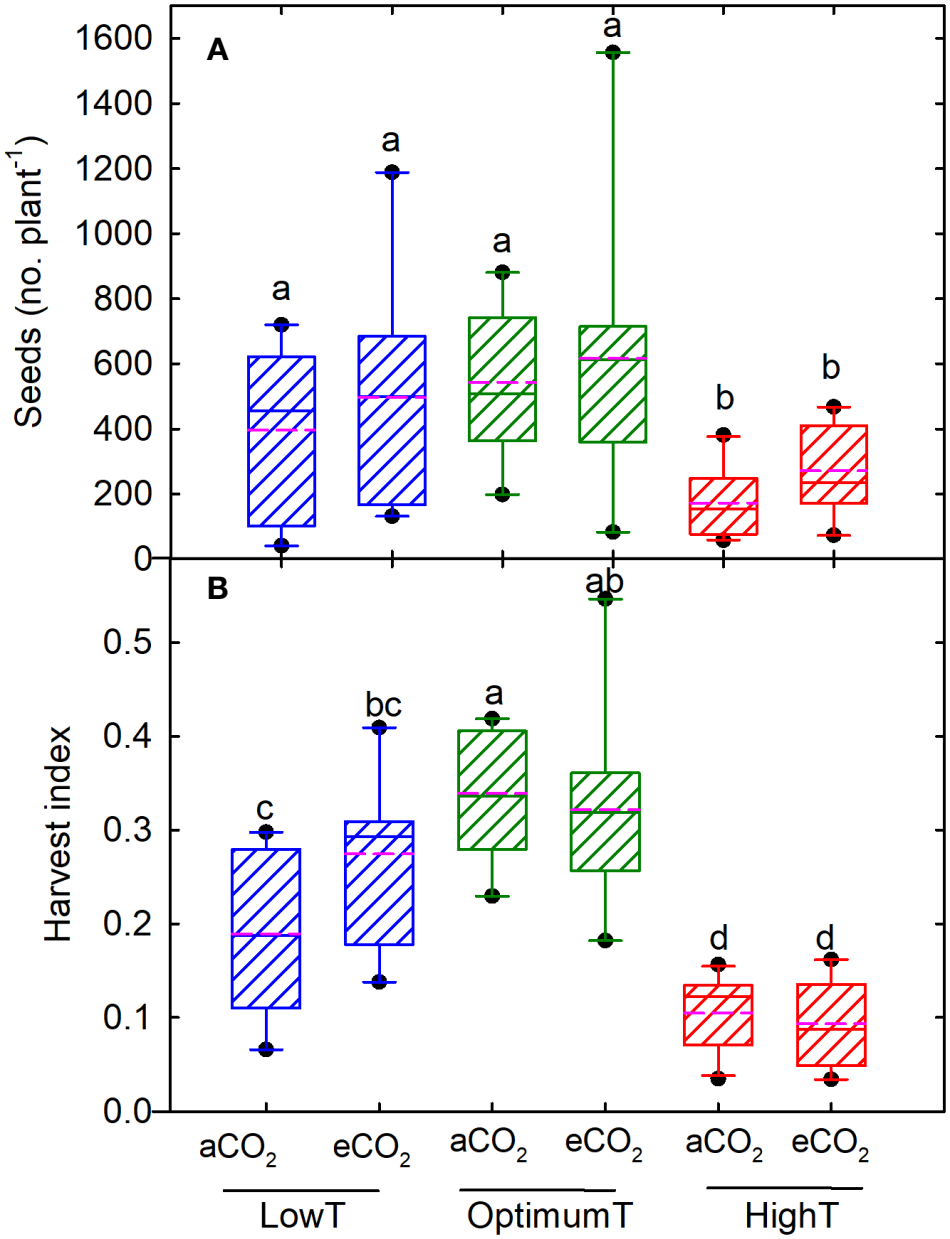
Temperature (LowT: 22/14°C; optimumT: 30/22°C; highT: 38/30°C) and CO_2_ (aCO_2_: 420 ppm and eCO_2_: 720 ppm) effects on **(A)** seed number, and **(B)** harvest index across DS25-1 DS31-243. The pink dotted line indicates the average values. The letters above the boxes are Duncan’s multiple range test (DMRT) between six treatments. The treatment boxes with similar letters are statistically non-significant.

In DS25-1, the maximum number of pods was observed under optimum temperature at eCO_2_ (347), followed by the low temperature at eCO_2_ (342) (**Figures 2A, B**). The eCO_2_ had a positive effect on the number of pods in both cultivars under high-temperature treatment, resulting in a pod number that was on par with the optimum and low-temperature conditions. The least number of pods was observed at high-temperature treatment and aCO_2_ in both cultivars. Comparatively, DS25-1 had a higher pod number in all the treatment conditions. Maximum seed number was observed at optimum temperature (606), and high temperature retarded the production of seeds (**Figure 3A**). A reduction of 68% and 54% in seed number was observed due to high temperature at aCO_2_ and eCO_2_, respectively, compared to optimum temperature and aCO_2_. The eCO_2_ did not result in any significant change in seed number. Under high temperatures, the eCO_2_ yielded a 30% but non-significant increase in seed numbers compared to aCO_2_. The harvest index of the cultivars declined with extreme temperature conditions exhibiting maximum harvest index at optimum temperature and aCO_2_ (0.34) followed by eCO_2_ (0.32) (**Figure 3B**). The high temperature resulted in a reduction of 70% harvest index under both aCO_2_ and eCO_2_, compared to the optimum temperature at aCO_2_. Additionally, even low temperatures declined the harvest index of the plant, which reflects the reduction in seed yield.

### 3.2 Temperature and CO_2_ effect on seed quality

**Figure 4:**
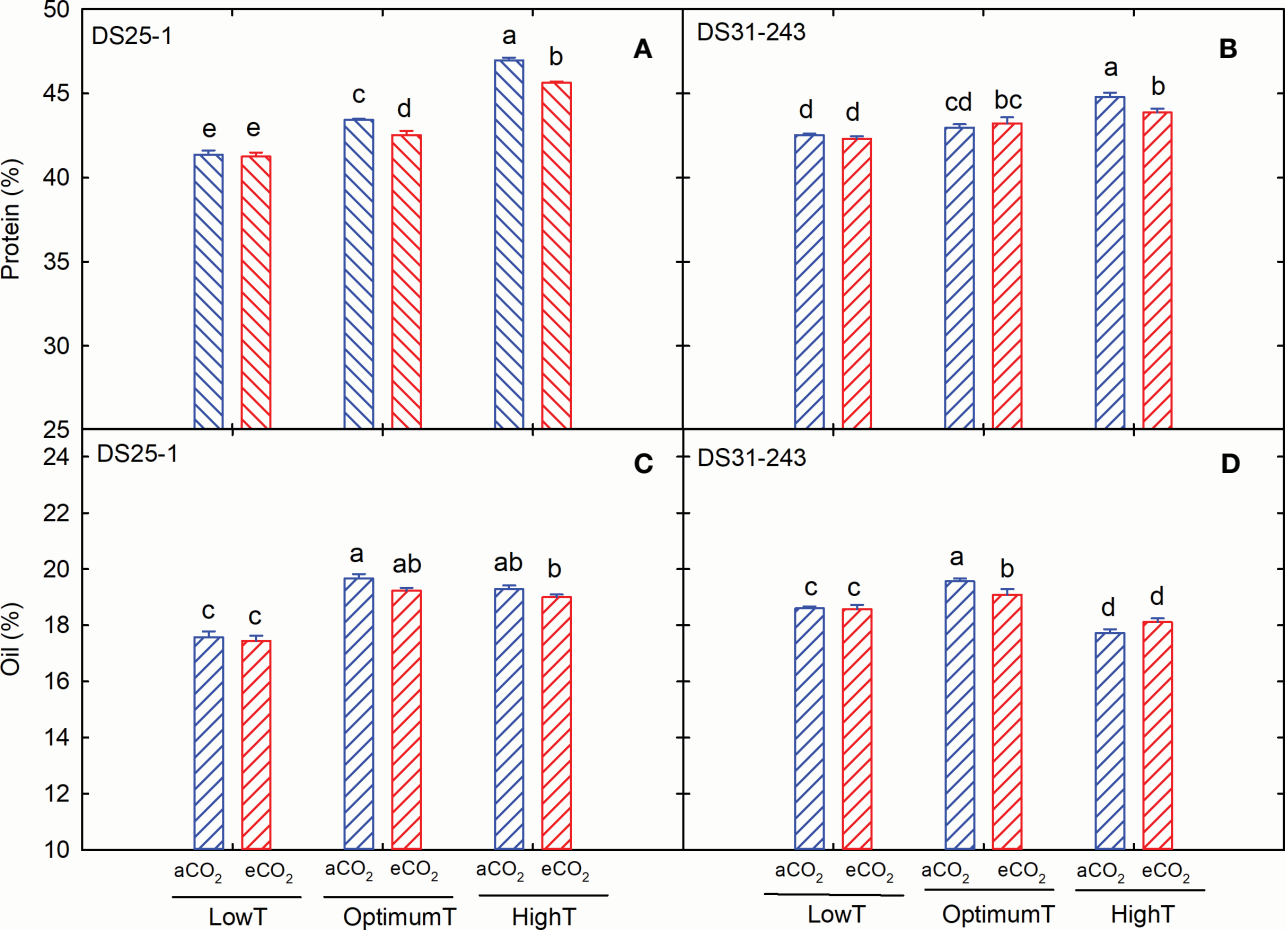
Temperature (LowT: 22/14°C; optimumT: 30/22°C; highT: 38/30°C) and CO_2_ (aCO_2_: 420 ppm and eCO_2_: 720 ppm) effects on **(A)** protein (%) of DS25-1 and **(B)** DS31-243, and **(C)** oil (%) of DS25-1 and **(D)** DS31-243. Bars represent the mean ± SE. The letters above the boxes are Duncan’s multiple range test (DMRT) between six treatments. The treatment boxes with similar letters are statistically non-significant.

The growing temperature had a remarkable effect on the seed chemical composition (**Table 2**), while the eCO_2_ only affected the accumulation of protein (p<0.001) and stachyose (p<0.05). The cultivars differed for protein, starch, stachyose, and oleic acids at p<0.05 to p<0.001. The interactions of these three main factors also resulted in significant changes in starch, stachyose, and linolenic acids (**Table 2**). In both cultivars, the increase in growing temperature during the flowering and seed development stages increased the seed protein content (**Figures 4A, B**). At low temperatures, eCO_2_ did not result in any detectable change in the protein, while at high temperatures, a 3% and 2% reduction in protein content was recorded for DS25-1 and DS31-243, respectively, compared to aCO_2_. In contrast, the maximum oil content was observed at optimum temperature and aCO_2_ in DS31-243 (19.5%) (**Figure 4D**). In the case of DS25-1, the oil content at optimum and high temperatures did not significantly differ at their respective CO_2_ environment (**Figure 4C**). On the other hand, the lowest oil content was recorded at low temperature and aCO_2_ (17.4%). In comparison, in DS31-243, it was recorded at high temperature and aCO_2_ (17.7%). The eCO_2_ did not significantly affect the oil content at low and high temperatures in DS31-243.

**Table 2:** Analysis of variance across temperature (T), CO_2,_ and cultivars (V) for seed quality parameters (*, **, *** represent significance level p ≤ 0.05, 0.01, and 0.001, respectively, and NS - non-significant).

Parameters	Protein	Oil	Sucrose	Starch	Stachyose	Oleic acid	Linoleic acid	Linolenic acid
**T**	***	***	***	***	***	***	***	***
**CO_2_**	***	NS	NS	NS	*	NS	NS	NS
**V**	*	NS	NS	***	*	***	NS	NS
T*CO_2_	**	NS	NS	***	***	**	**	NS
T*V	***	***	***	NS	NS	*	**	***
CO_2_*V	NS	NS	*	NS	NS	NS	NS	*
T*CO_2_*V	NS	NS	NS	*	**	NS	NS	*

**Figure 5:**
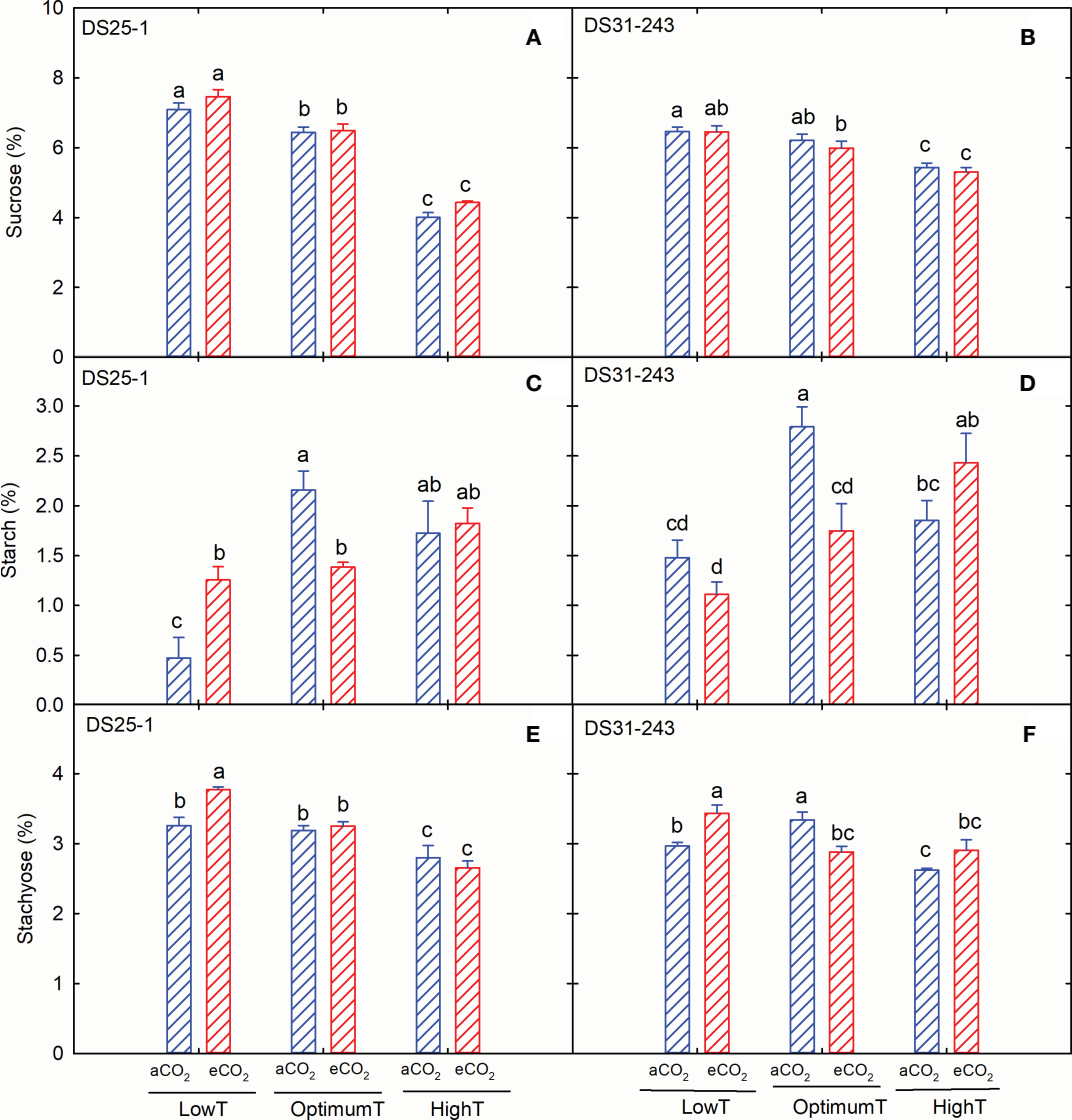
Temperature (LowT: 22/14°C; optimumT: 30/22°C; highT: 38/30°C) and CO_2_ (aCO_2_: 420 ppm and eCO_2_: 720 ppm) effects on **(A)** sucrose (%) of DS25-1 and **(B)** DS31-243, **(C)** starch (%) of DS25-1 and **(D)** DS31-243, and **(E)** stachyose (%) of DS25-1 and **(F)** DS31-243. Bars represent the mean ± SE. The letters above the boxes are Duncan’s multiple range test (DMRT) between six treatments. The treatment boxes with similar letters are statistically non-significant.

Sucrose content decreased with an increase in temperature in both cultivars (**Figures 5A, B**), but eCO_2_ did not significantly affect the sucrose accumulation. A reduction of 43% and 15% in sucrose content was recorded in DS25-1 and DS31-243, respectively, due to an increase in temperature from low to high at aCO_2_. On the other hand, the maximum starch content was observed at optimum temperature and aCO_2_ (**Figures 5C, D**) for both cultivars. Under extreme temperatures, eCO_2_ increased the starch content, except for DS31-243, at low-temperature treatment. However, at optimum temperature, eCO_2_ resulted in reduced starch accumulation compared to aCO_2_. Unlike starch accumulation, maximum accumulation of stachyose content was observed in low temperatures at eCO_2_ (**Figures 5E, F**). Like sucrose accumulation, the increase in temperature reduced the production of stachyose in seeds.

**Figure 6:**
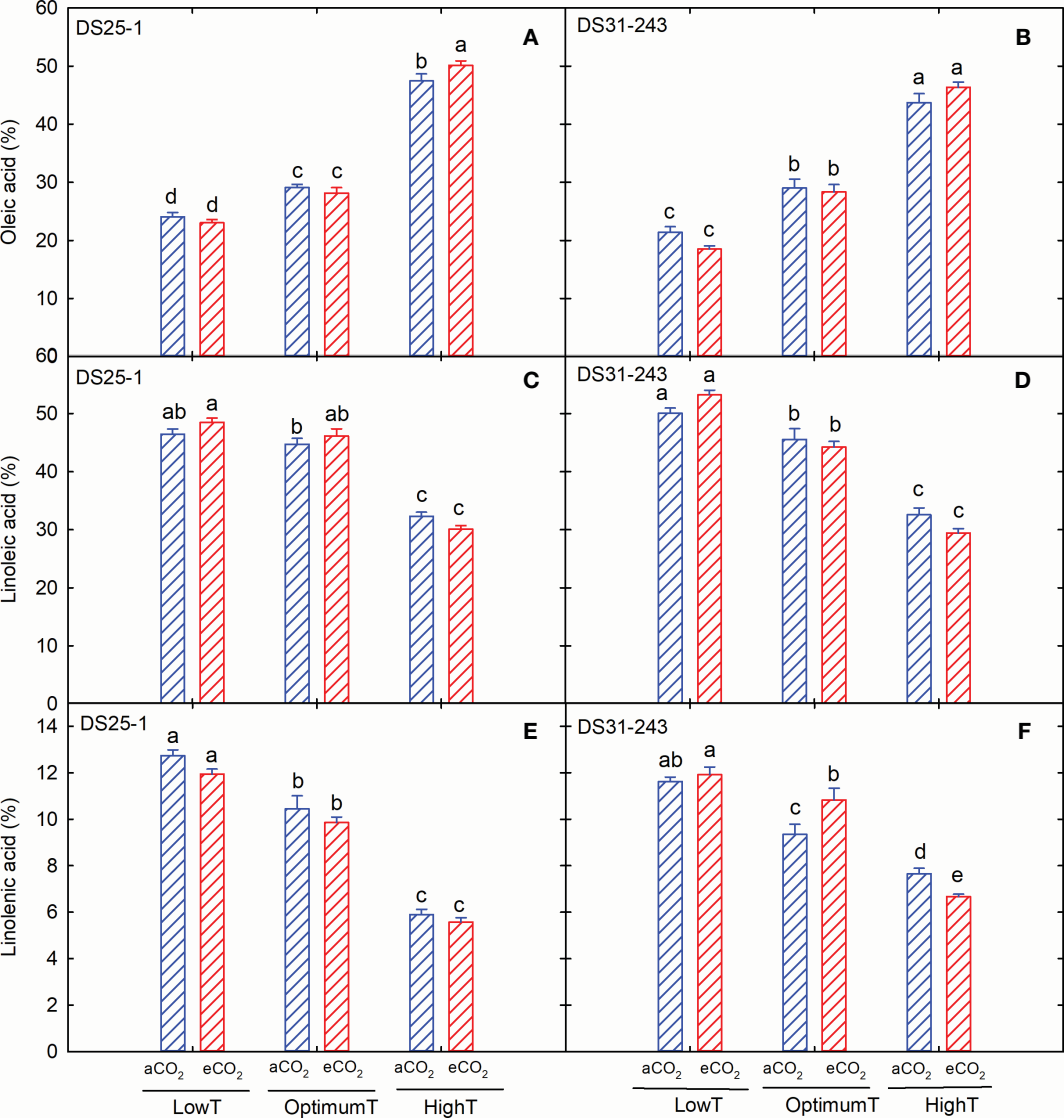
Temperature (LowT: 22/14°C; optimumT: 30/22°C; highT: 38/30°C) and CO_2_ (aCO_2_: 420 ppm and eCO_2_: 720 ppm) effects on **(A)** oleic acid (%) of DS25-1 and **(B)** DS31-243, **(C)** linoleic acid (%) of DS25-1 and **(D)** DS31-243, and **(E)** linolenic acid (%) of DS25-1 and **(F)** DS31-243. Bars represent the mean ± SE. The letters above the boxes are Duncan’s multiple range test (DMRT) between six treatments. The treatment boxes with similar letters are statistically non-significant.

Among the fatty acids, oleic acid content increased with an increase in growing temperature without being affected by the eCO_2_ concentration, except for DS25-1 at high temperatures (**Figures 6A, B**). The highest oleic acid content of 50% (DS25-1) and 46% (DS31-243) was observed in seeds grown under high temperatures and eCO_2_. Unlike oleic acid, linoleic and linolenic acid content decreased with an increase in temperature (**Figures 6C–F**). The maximum linoleic acid production was observed in seeds developed under low-temperature treatment, while eCO_2_ did not significantly influence the linoleic acid content in both cultivars. The increase in temperature from low to high at aCO_2_ resulted in a 30% and 35% decrease in linoleic acid content in DS25-1 and DS31-243, respectively. Similarly, linolenic acid content declined by 54% and 34%, respectively, in DS25-1 and DS31-243 under aCO_2_.

### 3.3 Temperature and CO_2_-induced transgenerational changes in seedling emergence and performance

**Figure 7:**
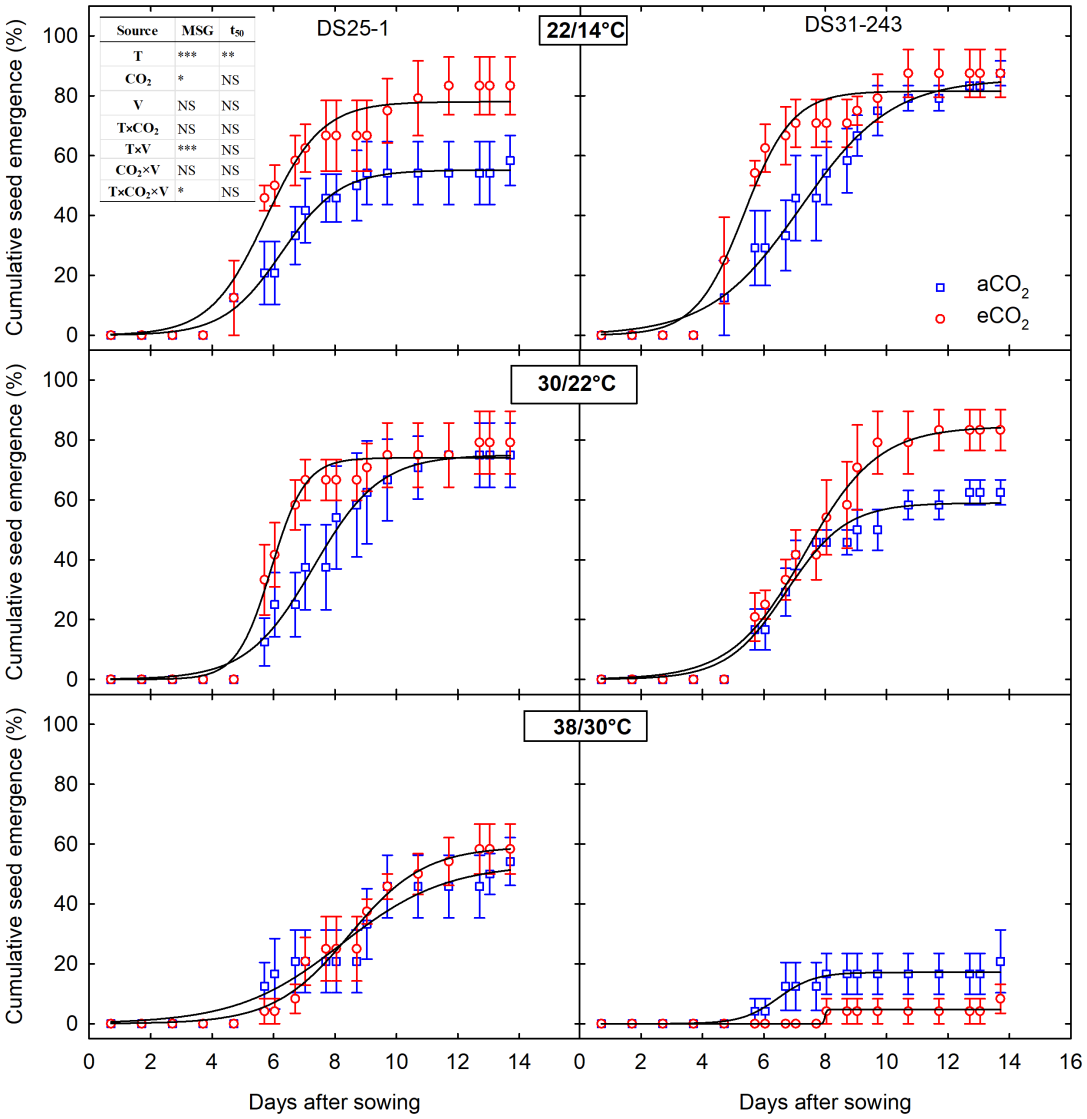
Temperature (LowT: 22/14°C; optimumT: 30/22°C; highT: 38/30°C) and CO_2_ (aCO_2_: 420 ppm and eCO_2_: 720 ppm) effects on cumulative seed emergence of progenies of two soybean cultivars (V; DS25-1 and DS31-243). The effect of temperature, CO_2_, cultivar, and their interactions are given in the table (*, **, *** represent significance level p ≤ 0.05, 0.01, and 0.001, respectively, and NS - non-significant).

The seedling emergence and biomass of progenies were significantly affected (p<0.001 to 0.5) by parental growing conditions (**Figure 7**; **Table 3**). The three-way interaction of temperature, CO_2_, and cultivar was non-significant for all the measured progeny parameters except for MSG (p<0.05) (**Table 3**). The time to 50% emergence was only affected by the parental temperature (p<0.01). The genetic difference in cultivar (p<0.01) contributed to the change in root-to-shoot ratio, and it was neither influenced by parental temperature nor CO_2_.

**Table 3:** Analysis of variance across temperature (T), CO_2,_ and varieties (V) for various parameters measured (*, **, *** represent significance level P ≤ 0.05, 0.01, and 0.001, respectively, and NS - nonsignificant).

Parameters	T	CO_2_	V	T*CO_2_	T*V	CO2*V	T*CO_2_*V
Plant height	NS	*	**	*	NS	*	NS
Leaf area	***	**	*	*	NS	***	NS
Shoot biomass	***	**	NS	*	NS	***	NS
Root biomass	**	NS	NS	NS	NS	*	NS
Total biomass	***	*	NS	*	NS	**	NS
Root-to-shoot ratio	NS	NS	**	NS	NS	NS	NS

**Figure 8:**
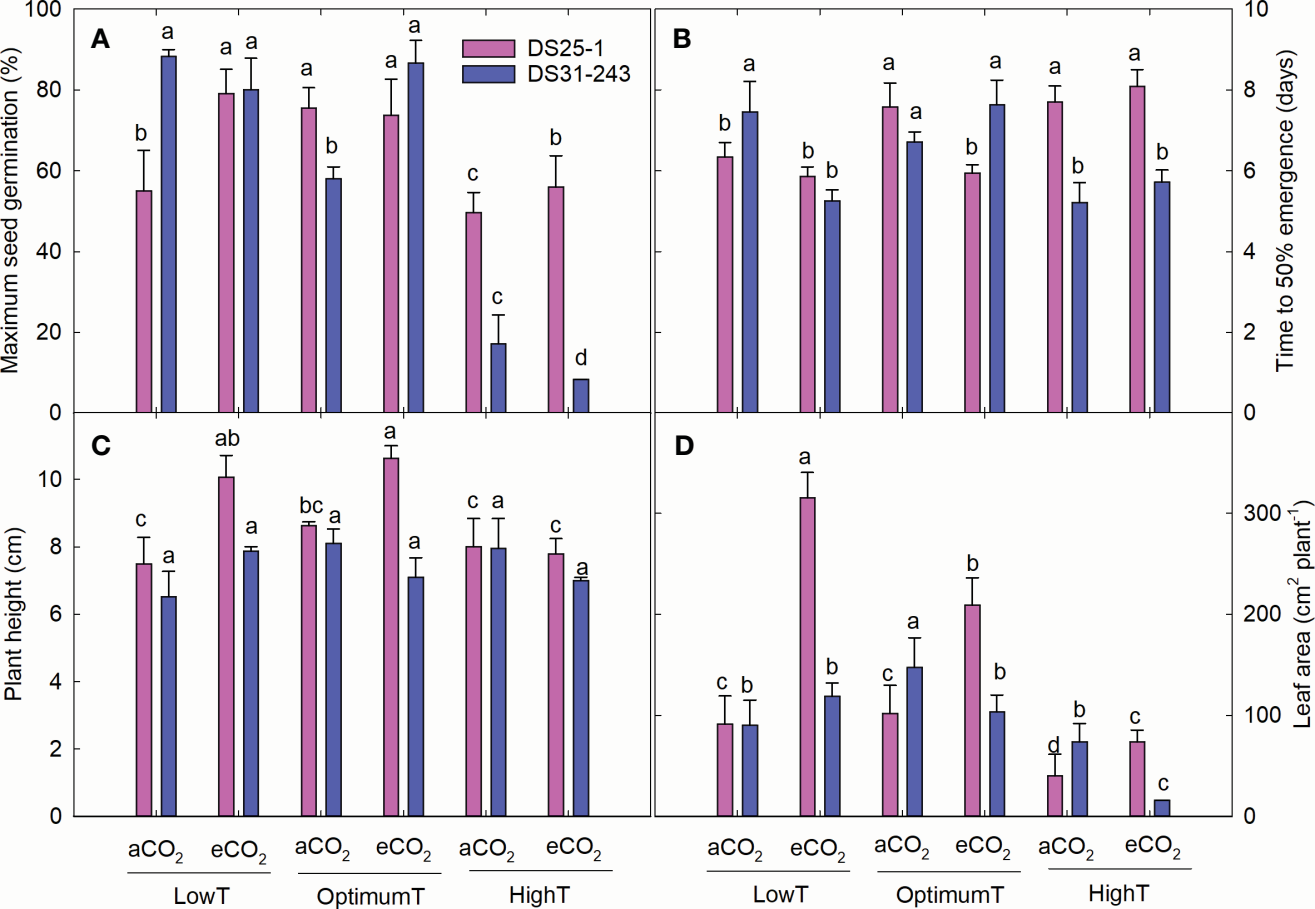
Transgenerational effect of temperature (LowT: 22/14°C; optimumT: 30/22°C; highT: 38/30°C) and CO2 (aCO_2_: 420 ppm and eCO_2_: 720 ppm) on **(A)** maximum seed germination, **(B)** time to 50% emergence, **(C)** plant height, and **(D)** leaf area of two cultivars of soybean. Bars represent the mean ± SE. The letters above the boxes are Duncan’s multiple range test (DMRT) between six treatments in each variety. The treatment boxes with similar letters are statistically non-significant.

Soybean seeds developed under high-temperature conditions had lower MSG of 49.6% at aCO_2_ and 56% at eCO_2_ for DS25-1. In comparison, it was 17% and 8.4%, respectively, for DS31-243 (**Figures 7**, **8A**). Highest MSG was recorded at parental low temperature for DS25-1 (79.1%) and DS31-243 (88.25%) at eCO_2_ and aCO_2_, respectively. The seeds produced at high temperatures took more time to reach 50% emergence of their MSG in DS25-1 (**Figure 8B**). Seeds grown under low parental temperature had quicker seed emergence than optimum parental temperature. DS25-1 seeds grown under high parental temperature took 7.7 and 8.1 days to reach 50% emergence in comparison with 6.34 and 5.86 days at low parental temperatures under aCO_2_ and eCO_2_, respectively. At high parental temperatures, DS31-243 took 5.21 and 5.72 days to reach 50% emergence of 17.14% and 8.35% MSG, respectively, at aCO_2_ and eCO_2_.

**Figure 9:**
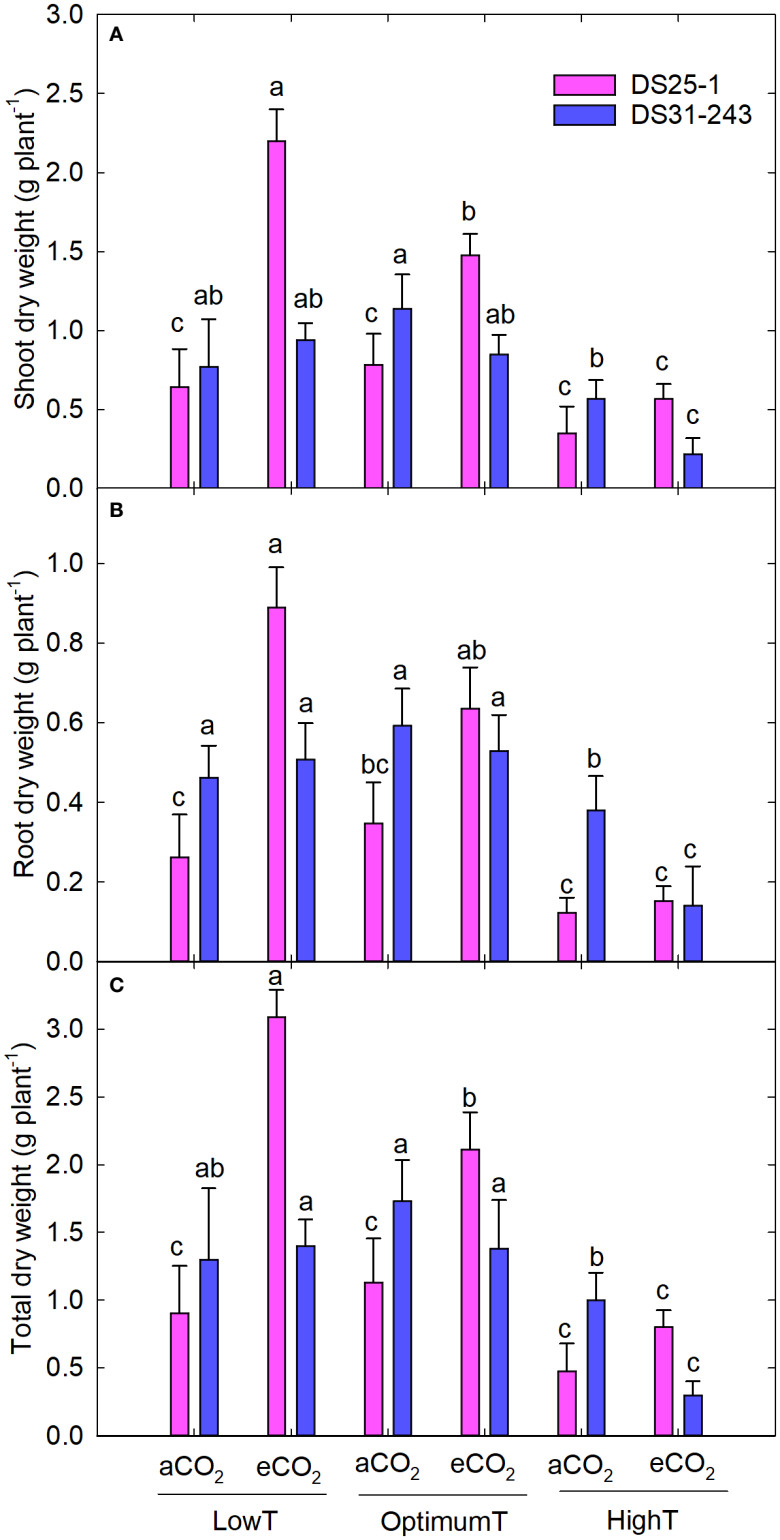
Transgenerational effect of temperature (LowT: 22/14°C; optimumT: 30/22°C; highT: 38/30°C) and CO2 (aCO_2_: 420 ppm and eCO_2_: 720 ppm) on **(A)** shoot dry weight, **(B)** root dry weight, and **(C)** total dry weight of two cultivars of soybean. Bars represent the mean ± SE. The letters above the boxes are Duncan’s multiple range test (DMRT) between six treatments in each variety. The treatment boxes with similar letters are statistically non-significant.

The parental eCO_2_ treatment increased the plant height of the seedlings in DS25-1 compared to seeds developed under aCO_2_ (**Figure 8C**). At the same time, the plant height in DS31-243 seedlings was not affected by the parental growing condition. A greater reduction in leaf area was noticed in seeds grown under parental high temperature, with a decrease of 61% and 64% under parental aCO_2_ and eCO_2_ in DS25-1, respectively, compared to the seeds grown under parental optimal temperature at aCO_2_ and eCO_2_ (**Figure 8D**). The maximum leaf area in DS25-1 was observed under parental low temperature and eCO_2_, while in DS31-243, it was observed in seeds grown under parental optimum temperature and aCO_2_. The eCO_2_ during the pod-filling stage at low and optimum temperatures increased the seedling biomass in DS25-1 (**Figure 9**). While seeds grown under eCO_2_ and high temperature did not influence the seedling biomass. Conversely, seeds harvested from plants exposed to eCO_2_ at optimum and high temperatures did not result in higher biomass accumulation in DS31-243. In DS25-1, the highest shoot biomass (2.2 g) was recorded from seeds produced under low temperature and eCO_2_ (**Figure 9A**). Similarly, the root (0.9 g) and total (3.1 g) biomass accumulation was higher in seeds developed under low temperature and eCO_2_ (**Figures 9B, C**). On the contrary, DS31-243 recorded the highest biomass accumulation (shoot: 1.1g; root: 0.6g; total: 1.7g) from seeds grown under parental optimum temperature and aCO_2_. The results suggest that the progeny performance is not only influenced by the growing environmental conditions of the parents but also governed by the genetic constitution of the cultivar.

**Figure 10:**
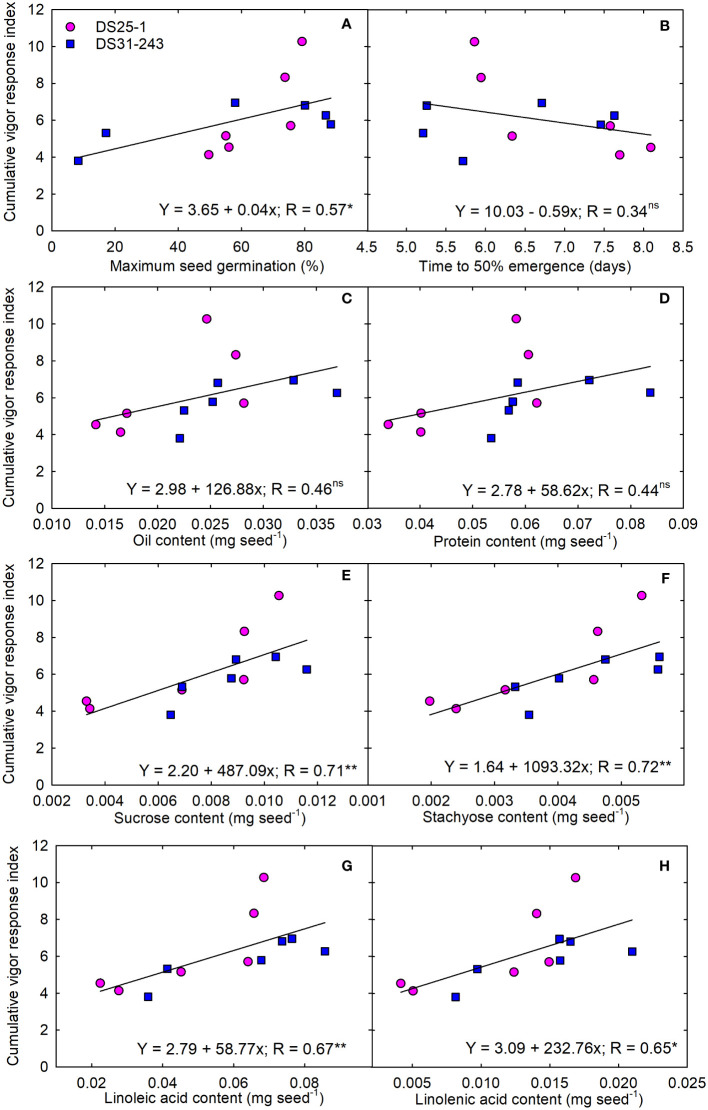
Linear regression between quality content per seed and cumulative vigor response index of progenies; **(A)** maximum seed germination (%), **(B)** time to 50% emergence (days), mg seed^-1^ content of **(C)** oil, **(D)** protein, **(E)** sucrose, **(F)** stachyose, **(G)** linoleic acid, and **(H)** linolenic acids. The * and ** indicate the statistical significance at p< 0.05 and 0.01. the ‘ns’ reference to nonsignificance.

To ascertain the role of seed chemical composition towards the seedling performance, we converted the measured seed quality components from percentage to milligrams per seed (mg seed^-1^). We observed a significant positive linear relationship between maximum seed germination and CVRI (R=0.57; p<0.05) (**Figure 10A**). While the time to 50% emergence had a negative slope and non-significant interaction with CVRI (**Figure 10B**). The relationship of both protein and oil content per-seed with CVRI was non-significant (**Figures 10C, D**). Among the carbohydrates measured in the study, stachyose content was highly positively correlated with CVRI (R= 0.72; p<0.01), followed by sucrose (R= 0.71; p<0.01) (**Figures 10E, F**). Among the fatty acids, both linoleic and linolenic acids had a positive linear relationship with CVRI having regression coefficient of R= 0.67 (p<0.01) and R= 0.65 (p<0.05), respectively (**Figures 10G, H**).

## 4 Discussion

The investigation shows a discernible shift in seed yield and quality attributed to the fluctuations in growing temperature during the flowering and seed development stages. The day/night temperatures above 30/22°C were observed to be detrimental to soybean seed production, hindering yield. The atmospheric CO_2_ concentration played a minimal role in influencing these traits, garnering attention from soybean growers and policymakers alike. The experiment also validated the transgenerational impact of temperature and CO_2_ on seed emergence and seedling vigor. We also observed that the degree of influence is intricately tied to the genetic composition of the seed. The following are the major findings of our study.

### 4.1 eCO_2_ did not compensate for the yield loss under high temperature

The high temperature during the flowering and seed-filling stage decreased the seed yield by 83% over the optimum temperature. The flowering stage is one of the most critical stages in the life cycle of soybean, and the temperature variation could instead lead to altered plant performance, affecting final yield (No et al., 2021; Poudel et al., 2023). It is also important to note that stress at the reproductive stage often has no chance of recovery, resulting in severe yield loss, unlike the vegetative stage (Jumrani and Bhatia, 2018). It has been reported that the optimum temperature for soybean vegetative growth is 30°C (Hesketh et al., 1973), while for anthesis, it is 26°C (Hatfield et al., 2011). The temperature stress at the reproductive stage can lead to flower abortion, primarily attributed to poor pollen germination and stigma receptivity, which impairs fertilization, leading to a lower number of fruit sets and pod filling (Siebers et al., 2015; Iovane and Aronne, 2022). Salem et al. (2007) reported a reduction of 34%, 56%, and 33% in pollen production, pollen germination, and pollen tube elongation, respectively, in soybean when grown under 38/30°C temperature compared to 30/22°C. These outcomes provide a clear explanation for our observation on the reduction in seed number by 68% and 58% under high temperatures of 38/30°C for both aCO_2_ and eCO_2_, respectively.

Plants accumulate dry matter through photosynthesis, which is an intricate process that is highly plastic to the surrounding environment. The lowered rate of photosynthesis and associated physiological activities of soybean under high temperatures above 30/22°C has resulted in a reduction in seed number and seed yield (Jumrani and Bhatia, 2018). Higher temperatures cause early leaf senescence (Hatfield and Prueger, 2015), leading to poor partitioning of photosynthate to the seed, especially when the plant is stressed during the seed developmental stage. The lower dry matter accumulation and imbalance in a source-sink relationship at high temperatures could result in a decreased harvest index (Rivelli et al., 2024). Since the cultivars under study were indeterminate growth types and the increase in canopy temperature accelerated the vegetative development, the plants put more energy into the number of nodes, compromising seed yield. The result was in agreement with the findings of Burroughs et al. (2023).

Like high temperature, low temperature also influences key physiological factors in plants. Under temperatures of 22/14°C and aCO_2_, the seed yield, 100 seed weight, pod number, and harvest index were reduced compared to plants grown under 30/22°C. However, the negative impact of high temperature was more severe than low temperature. It has been reported that temperatures below 25°C reduce growth and photosynthetic rate and further lowering disturbed the electron transport system in soybean (Szczerba et al., 2021). Our finding is also supported by Alsajri et al. (2022), displaying reduced stomatal conductance and photosynthesis at 17.3°C, which led to a 6% reduction in 100 seed weight and subsequent decrease in seed yield. Staniak et al. (2021) reported that the day/night temperature of 17/13°C during flowering negatively influenced the plant structure and reduced pod number and seed yield in soybean.

The eCO_2_ did not significantly influence most of the seed yield components. There are disparities in the response of C_3_ plants to eCO_2_. Soares et al. (2019) reported a considerable cultivar difference in seed yield (ranging from -23.8 to 39.6%) in response to eCO_2_ in soybean. It has been reported that eCO_2_ either increases the photosynthesis in non-nodulating soybean cultivars (Ainsworth et al., 2002) or the response to seed yield is less than the response of total biomass (Soares et al., 2019). At optimum temperature, the non-significant but slight decrease in harvest index under eCO_2_ compared to aCO_2_ could explain the increased total biomass and reduced seed yield in our study.

### 4.2 eCO_2_ did not alter the high temperature-induced changes in seed quality

The growing temperature at flowering and seed developmental stages altered the soybean seed quality. The accumulation of seed protein and oil gradually increases two weeks after fertilization (Sale and Campbell, 1980). Thus, an increase in temperature from 22/14°C to 38/30°C during this period impacted the concentration of seed protein and oil. The heat stress-induced production of enzymes and proteins as part of soybean heat stress tolerance (Kidokoro et al., 2015) has resulted in increased protein content in the cultivars. Being a heat-tolerant cultivar, DS25-1 had higher protein accumulation under high temperatures compared to DS31-243. However, the eCO_2_ resulted in a reduction of protein and oil content in seeds, attributed to the dilution effect as eCO_2_ slightly increases the carbohydrate content in seeds (Li et al., 2018). Across the cultivars and temperatures, the studied total carbohydrate content was slightly higher at eCO_2_ (2.7%) than at aCO_2_ (2.5%). Hampton et al. (2013) reported that the increase in seed carbon to nitrogen ratio under eCO_2_ resulted in a decrease in protein content compared to aCO_2_. Apart from temperature and eCO_2_, other growth factors such as water, nitrogen uptake, and photoperiod influence protein and oil accumulation in seeds. In this study, the plants were grown under optimum water and nutrient conditions in sunlit chambers without limiting protein accumulation. This resulted in an increased concentration of seed protein with an increase in temperature in both cultivars, which is in line with the outcomes of Alsajri et al. (2020) under similar experimental conditions. Unlike protein, the oil content decreased under high temperatures. Maximum oil content of 19% in both DS31-243 and DS25-1 was recorded at the optimum growth temperature. In contrast, low and high temperature reduced the oil accumulation, supporting the quadratic relationship reported by Alsajri et al. (2020) in soybean.

Sucrose is the major energy source for fermentation (Arendt and Zannini, 2013). High temperature reduced the sucrose content in DS31-243 and DS25-1 without being significantly affected by eCO_2_. This negative relationship between sucrose content and temperature was reported by Kumar et al. (2010), who reported higher sucrose content at 18/13°C. Decreased photosynthate allocation to seeds during the seed-filling stage at elevated temperatures resulted in reduced carbohydrate content. Given the heightened respiratory demand in elevated temperatures, the availability of oxygen becomes constrained, leading to anaerobic conditions. This limitation impedes phloem unloading from seed coat vascular bundles to the apoplast surrounding the cotyledon cells (Thorne, 1982). The greater accumulation of stachyose content at low temperatures aligns with the findings of Alsajri et al. (2020), reporting 55 mg g^-1^ of stachyose content at 21/13°C.

The linoleic and linolenic acids (omega-6 and omega-3 polyunsaturated fatty acids, respectively) decreased with an increase in growing temperature, while oleic acids increased. The results of the study align with the findings of Alsajri et al. (2020) and Bukowski and Goslee (2024). Notably, as the temperature increased from 15°C to 40°C, there was a reduction in linoleic and linolenic concentrations from 55% and 13% to 30% and 3.5%, respectively (Bukowski and Goslee, 2024). The alteration in nitrogen and carbon fixation and metabolic pathways led to modification in the polyunsaturated fatty acids (PUFA) in soybeans (Bellaloui et al., 2013). It has been reported that high temperatures above 30-35°C decrease linoleate desaturase activity and degrade the omega-6 desaturase enzyme encoded by the FAD2-1A gene (Cheesbrough, 1989). The high levels of FAD2-2C isoforms of GmFAD2 at 18/12°C (Schlueter et al., 2007) explain the high levels of linoleic acid observed under low temperatures in this study. In addition, the FAD3 enzyme, involved in the production of PUFA, was observed to degrade under temperatures above 30°C (O’Quin et al., 2010). Interestingly, the decreased PUFA (linoleic and linolenic acids) and increased monounsaturated fatty acids, in other words, decreased unsaturation, under high temperature during early seed developmental stages is a heat tolerance mechanism to ensure membrane stability to protect cell function and embryo development and prevent fatty acid oxidation (Nadakuduti et al., 2023). The eCO_2_ did not affect the accumulation of fatty acid except for linolenic acid in DS31-243. The reduced sucrose content at high temperature decreased the production of pyruvate and adenosine triphosphate (ATP), which is involved in the production of fatty acids (Andre and Benning, 2007). Additionally, the high energy requirement for the production of polyunsaturated fatty acids (linoleic and linolenic acids) than monounsaturated fatty acids (oleic acid) resulted in higher accumulation of oleic acid during seed filling stage at higher temperature.

### 4.3 Temperature and eCO_2_ exhibit a transgenerational effect on progenies

The performance of progenies was significantly affected by the parental growing conditions. The higher MSG of DS25-1 compared to DS31-243 at high parental temperature confirms the heat-tolerance characteristics of the cultivar. High-temperature stress prior to the physiological maturity of developing seeds has the potential to reduce germination (Alsajri et al., 2022). This is attributed to the inhibition of the plant’s ability to supply necessary assimilates crucial for synthesizing storage compounds essential for successful germination (Hampton et al., 2013). Sparse evidence indicates that only brief episodes of high-temperature stress during critical seed development stages may be adequate to diminish seed vigor (Begcy et al., 2018). A deficient resource supply to the seeds, combined with premature senescence, has adverse effects on seed weight and the composition of seed quality (Alsajri et al., 2022). This leads to a constrained supply of resources from cotyledon to the developing seedlings, resulting in a reduced accumulation of biomass, as evident in the study.

Genotype-by-environment interactions create intraspecific variation in plant responses to environmental treatments. Natural selection acts on this evolving trait plasticity adapted to local conditions influenced by past environments (Groot et al., 2017). The transgenerational plasticity can be either physiologically controlled or epigenetic or by the mother plant through progeny seed coat or endosperm modifications (Suter and Widmer, 2013; Deng et al., 2021). The transgenerational effect will be more pronounced when the progenies are subjected to parental environmental conditions (Uller, 2008). Despite assessing the progenies under standard growth conditions, our study unveiled the transgenerational memory of high parental temperatures, evidenced by the progenies exhibiting reduced MSG and seedling vigor. Transgenerational memory is retained through chromatin modifications, small RNA, and metabolite changes in the reproductive structures (Bilichak and Kovalchuk, 2016). The heat-induced regulated cell death, such as necrosis, apoptosis, and ferroptosis pathways, selectively eliminate specific cells in tissues to uphold homeostasis (Distéfano et al., 2021). This might lead to detectable changes in subsequent generations. Many heat stress-induced DNA methylation and histone changes are reported by several studies (Ramakrishnan et al., 2022; Cao and Chen, 2024). The environment-induced alteration in non-coding RNAs like micro RNAs (miRNA) and small-interfering RNAs (siRNAs) has been reported to play a regulatory role in epigenetic phenomena (Liu et al., 2016). Heat stress-induced modification in miRNA was reported to affect seed storage reserves by altering enzymes such as sucrose synthase, beta-glucosidase, and starch synthase in wheat (Liu et al., 2020). The heat stress-induced expression of Lysine-specific histone demethylase-1 (LSD1), which is involved in the demethylation of histone H3 lysine 4 has been reported in heat-stressed progenies in wheat (Wang et al., 2016). In addition, environmental changes can destabilize the genome either by mobilizing transposable elements or by increasing homologous recombination (Migicovsky and Kovalchuk, 2013). The disruption of the *MutS HOMOLOG 1* (*MSH1*) gene, which encodes a homolog of bacterial mismatch repair protein involved in organelle genome stability, is reported to have increased recombination of repeated sequences, induces male sterility, dwarfed growth, and delayed flowering under stressful environmental conditions in several plants (Ou et al., 2012; Bilichak and Kovalchuk, 2016). The impact of eCO_2_ was notably significant across various seedling vigor traits, indicating that even a slight gain in seed composition per seed could lead to discernible changes in seedling characteristics. Panda et al. (2023) reported the transgenerational effect of eCO_2_ on *A. thaliana* and *Physcomitrium patens* and demonstrated heritable DNA methylation changes due to eCO_2_. Shi et al. (2016) and Begcy et al. (2018) reported that high temperature modifies the quantity and sensitivity of phytohormones and enzymes in seeds, influencing the subsequent generation of seed germination. However, the exact genetic and epigenetic mechanism underlying the transgenerational effect of environmental factors is not yet detailed in soybeans. Our study was only confined to studying the visible changes brought by the parental growing conditions.

### 4.4 Single-seed chemical composition determines the seedling performance

Post-germinative growth preceding the plant’s attainment of photosynthetic self-sufficiency is mainly propelled by the utilization of seed reserves initially stored in the cotyledons of the dormant embryo (Bradow and Bauer, 2010). These dynamics significantly shape the plant’s initial stages of development (Snider et al., 2020). The CVRI was positively associated with MSG, sucrose, stachyose, and polyunsaturated fatty acids. Sugars are an essential source of energy, acting as osmotic solutes, building blocks, and signaling molecules (Ruan, 2014). During seed germination and early seedling growth, sucrose acts as an energy source for seed germination upon imbibition. Studies by Snider et al. (2014, 2016) revealed that oil content (mg seed^-1^) and total oil + protein (kcal seed^-1^) strongly and positively influenced the seedling vigor in cotton. However, our study resulted in a significant relationship between seedling vigor with carbohydrates and polyunsaturated fatty acids. It has been reported that the mobilization of lipids and the initiation of gluconeogenesis from lipid precursors contribute to carbohydrates that can be assimilated into the body of a developing seedling or employed in respiratory processes (Snider et al., 2020). Thus, increased carbohydrates and fatty acids improve the seedling growth and performance.

## 5 Conclusion

The study delineated the optimal temperature requirements (30/22°C) of soybean during its flowering and seed-filling stages to achieve maximum yield. Exposure of plants to a high temperature of 38/30°C significantly reduced all the seed yield components in both cultivars and the eCO_2_ did not consistently compensate for the yield loss. The seed yield was also affected by low temperatures, while the severity increased with an increase in temperature from optimum, resulting in an average reduction of 83%. The deviations in temperature from the optimum levels during flowering and seed-filling stages significantly altered the seed′s chemical composition. Notably, the protein content increased with rising temperatures while the oil content dropped.

Conversely, high temperatures hindered the accumulation of sucrose and stachyose in seeds without being significantly affected by the eCO_2_. Similarly, the polyunsaturated fatty acids decreased while oleic acid content rose under high temperatures. The cultivars exhibited a promising difference in their response to temperature and eCO_2_ treatments, explaining the genetic variation at play. A considerable transgenerational effect of parental exposure to high temperatures and eCO_2_ was observed on the emergence and growth of progenies, emphasizing the long-term implications of climate change on soybean. Seeds developed under 38/30°C temperature had lower MSG, prolonged t_50_, and reduced biomass accumulation. However, the extent of the effect was determined by the genetics of the cultivar. The eCO_2_ increased the seedling performance across the parental temperatures in DS25-1, while the DS31-243 did not exhibit this trend. In addition, a notable positive association was found between the per-seed chemical composition of carbohydrates and fatty acids with seedling vigor. Nonetheless, further research needs to be conducted to validate the association of seed composition with seedling vigor transition.

## Data availability statement

The original contributions presented in the study are included in the article/**Supplementary Material**. Further inquiries can be directed to the corresponding author.

## Author contributions

NT: Writing – review & editing, Writing – original draft, Visualization, Software, Formal analysis. RB: Writing – review & editing, Conceptualization. KNR: Writing – review & editing, Funding acquisition. WG: Writing – review & editing. KRR: Writing – review & editing, Writing – original draft, Visualization, Validation, Supervision, Software, Resources, Project administration, Methodology, Investigation, Funding acquisition, Formal analysis, Data curation, Conceptualization.
