# Supplementary material for: Temperature and elevated CO2 alter soybean seed yield and quality, exhibiting transgenerational effects on seedling emergence and vigor
Source: Front Plant Sci. 2024 Jul 31;15:1427086. doi: 10.3389/fpls.2024.1427086 (PMC11322351; doi:10.3389/fpls.2024.1427086)
Supplement: Supplementary file 1 [file DataSheet_1.docx]

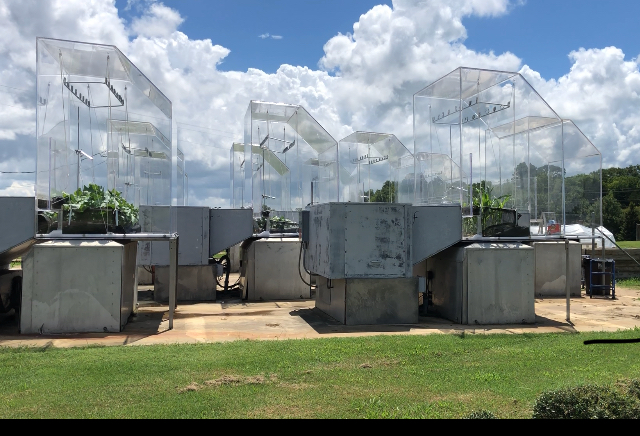


Supplementary Fig. 1. The Soil-Plant-Atmosphere-Research facility (SPAR) used in the study is located at the Environmental Plant Physiology Laboratory, Mississippi State University, Mississippi, MS, USA. Each SPAR facility consists of a plexiglass chamber to hold the plant canopy and a metal soil bin to accommodate the root system. A heating and cooling system at the rear of the facility controls the temperature inside the chamber. A CO_2_ analyzer monitors and adjusts the CO_2_ levels.
